# MrMYB44-Like Negatively Regulates Anthocyanin Biosynthesis and Causes Spring Leaf Color of *Malus* ‘Radiant’ to Fade From Red to Green

**DOI:** 10.3389/fpls.2022.822340

**Published:** 2022-02-01

**Authors:** Jia-Xin Meng, Jun Wei, Ru-Fei Chi, Yu-Hang Qiao, Jing Zhou, Yi-Lin Wang, Han Wang, Hou-Hua Li

**Affiliations:** College of Landscape Architecture and Art, Northwest A&F University, Yangling, China

**Keywords:** anthocyanin, transcriptome, *MYB44-like*, *WRKY6*, spring leaf color, transcription factors, crabapple

## Abstract

The “Spring-red-leaf” crabapple cultivar has young red leaves and mature green leaves. However, the mechanism of anthocyanin biosynthesis in crabapple leaves in spring remains unknown. In this study, Illumina RNA sequencing (RNA-Seq) was performed on *Malus* ‘Radiant’ leaf tissues in different stages of development. Twenty-two genes in the anthocyanin biosynthesis pathway and 44 MYB transcription factors (TFs) were significantly enriched among differentially expressed genes (DEGs). Three R2R3-MYB TFs in subgroup 22 of the MYB TF family, *MrMYB44-like1*, *MrMYB44-like2*, and *MrMYB44-like3*, were highly expressed in green leaves according to RNA-Seq and quantitative real-time quantitative PCR results. Their expression levels were negatively correlated with anthocyanin content. In transient assays, overexpression of *MrMYB44-like1*, *MrMYB44-like2*, or *MrMYB44-like3* inhibited anthocyanin accumulation and reduced pigment in leaf disks of *M*. ‘Radiant’ and fruit peels of *M. domestica* ‘Fuji.’ When the conserved region of the three *MrMYB44-like*s was silenced, the anthocyanin biosynthesis pathway was activated and pigments increased in both tissues. Moreover, bimolecular fluorescence complementation assays showed *MrMYB44-like*s interacted with *MrWRKY6* to form protein complexes that regulated anthocyanin biosynthesis.

## Introduction

*Malus* crabapple (*Malus* Mill.) is an ornamental deciduous tree or shrub in the family Rosaceae. It is a popular decorative plant in landscapes because of its pleasing form and attractive colors (Cui et al., 2018). *Malus* ‘Radiant’ is a “Spring-red-leaf” crabapple cultivar with young red leaves and green mature leaves, indicating complex changes in coloration. This quality trait is primarily determined by its metabolite composition (Yang et al., 2018).

Anthocyanins are the main pigments in flowers, leaves, and fruits and generate characteristic red, blue, and purple hues (Winkel-Shirley, 2001; Jaakola, 2013). Bright organ colors derived from anthocyanin accumulation directly determine the ornamental value of plants (Xie et al., 2012).

The anthocyanin metabolic pathway includes anthocyanin biosynthesis and degradation. Anthocyanin biosynthesis is derived from branches of the phenylalanine pathway (Koes et al., 2005; Petroni and Tonelli, 2011). Major enzymes include phenylalanine ammonia lyase (PAL), cinnamate-4-hydroxylase (C4H), 4-coumarate: CoA ligase (4CL), chalcone synthase (CHS), chalcone isomerase (CHI), flavanone 3-hydroxylase (F3H), flavonoid 3′-hydroxylase (F3′H), dihydroflavonol 4-reductase (DFR), anthocyanin synthase (ANS), and UDP-glucose: flavonoid 3-O-glu-cosyltransferase (UFGT). The enzymes encoded by the genes are responsible for biochemical reactions in anthocyanin biosynthesis (Honda et al., 2002). Anthocyanin degradation also affects color change. Loss of red pigmentation may be caused by increases in actively induced anthocyanin degradation, as well as termination of anthocyanin biosynthesis and dilution by growth (Oren-Shamir, 2009). Enzymes in three common families were recently found to participate in anthocyanin degradation, including β-glucosidase (BGLU), polyphenol oxidase (PPO), peroxidase (PER), and laccase (LAC) (Ying et al., 2018).

Expression of genes in the anthocyanin biosynthesis pathway is primarily regulated by a series of transcription factors (TFs). The MYB family of TFs is important in the anthocyanin biosynthesis pathway because it activates or inhibits genes by directly or indirectly binding the *cis*-acting element of DNA (Chen L. et al., 2019). As activators, MYB TFs usually form protein complexes with bHLH and WD40 (Albert et al., 2011). For example, the Aft (MYB) protein interacts with SlJAF13 (bHLH) and SlAN11 (WDR) forming an MBW complex and enhancing anthocyanin content in tomato (*Solanum lycopersicum*) (Yan et al., 2020). There has been a recent notable focus on MYB repressors (Ma and Constabel, 2019). The MYB repressors can act directly on promoters of structural genes (Yan et al., 2021), like *NtMYB3* represses promoter activity of *NtFLS* in Chinese narcissus (*Narcissus tazetta* L. var. chinensis) (Anwar et al., 2019) and *MdMYB6* inhibits the promoter activity of *MdANS* and *MdGSTF12* in apple (*Malus domestica*) (Xu et al., 2020). They can also passively repress anthocyanin biosynthesis by interacting with bHLH proteins to compete with MYB activators in the MYB–bHLH complex, thereby reducing its activation capability. For example, *MdMYB15L* interacts with *MdbHLH33* in *Malus* (Xu et al., 2018); *VvMYB4b* interacts with *VvMYC1* (bHLH) in *Vitis vinifera* (Cavallini et al., 2015); and *MtMYB2* interacts with *MtTT8* (bHLH) in *Medicago truncatula* (Jun et al., 2015).

Moreover, other TF families also regulate anthocyanin biosynthesis *via* interaction with MYB TFs, like *AtSPL9*, negatively regulates anthocyanin accumulation through destabilization of a MYB-bHLH-WD40 transcriptional activation complex in *Arabidopsis* (Gou et al., 2011); *PyWRKY26* interacts with *PybHLH3* cotarget the *PyMYB114* promoter resulting in anthocyanin accumulation in red-skinned pear (*Pyrus* L.) (Li et al., 2020); *IbNAC56a* and *IbNAC56b* interact with *IbMYB340* and *IbbHLH2* to positively regulate anthocyanin biosynthesis by binding to the *IbANS* promoter in sweet potato (*Ipomoea batatas*) (Wei et al., 2020).

Fading color in spring leaves is common, but for crabapple, the mechanism of changes in leaf coloration in natural conditions remains unclear. In this study, contents of anthocyanin metabolites and expression of differentially expressed genes (DEGs) in young red and green mature leaf tissues of *M*. ‘Radiant’ were analyzed to determine the key genes involved in anthocyanin biosynthesis. Then, the function of candidate key TFs was determined. This study increases understanding of the mechanism of pigmentation underlying color changes in leaves and therefore will facilitate breeding plants with desirable color traits.

## Materials and Methods

### Plant Material

**FIGURE 1:**
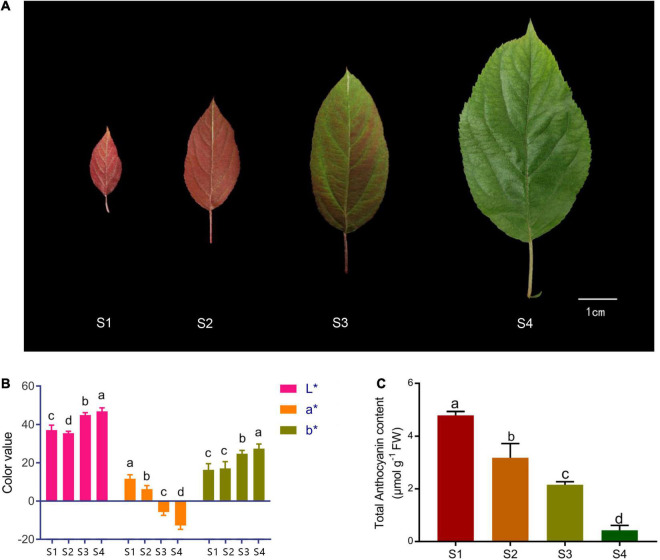
Phenotype and total anthocyanin content at different stages of spring leaf development in *Malus* ‘Radiant.’ **(A)** Leaf color at four stages (S1–S4). **(B)** L*, a*, and b* values of leaves at four stages. **(C)** Total anthocyanin content at four stages (FW, fresh weight). Error bars represent the standard errors. Different lowercase letters indicate significant differences among treatments according to one-way ANOVA test (*P* < 0.05).

*Malus* ‘Radiant’ plants were grown in the crabapple germplasm nursery of Northwest A&F University, Yangling, China, under natural conditions. During growth, leaf phenotype gradually changes during development and can be divided into four stages (S1–S4, Figure 1A). Tissue samples were immediately frozen in liquid nitrogen and stored at −80°C until RNA extraction and total anthocyanin and high-pressure liquid chromatography (HPLC) analyses.

### Leaf Color Measurement

Colors of fresh leaves were measured with a CR-400 chroma meter (Konica Minolta, Tokyo, Japan). Parameters L*, a*, and b* were determined. Parameter L* indicates lightness (ranging from 0 to 100). Positive and negative values of a* are standard for red and green, respectively, and those of b* represent yellow and blue, respectively (both ranging from −60 to +60) (Mcguire, 1992; Liu et al., 2016). Means were based on five independent biological replicates with three technological replicates.

### Measurement of Total Anthocyanin Contents

Total anthocyanins were extracted with a methanol–hydrochloric acid (HCL) solution (Lee and Wicker, 1991). Leaf samples (0.5 g) were incubated in 10 ml of methanol and 0.1% HCL (v/v) at 4°C for 48 h in the dark. Mixtures were centrifuged at 6,000 rpm for 3 min, and supernatants collected. Absorbance was measured at 530, 620, and 657 nm, in triplicate. Relative anthocyanin content was determined as follows:


A=(A530⁢nm-A620⁢nm)-0.1⁢(A650⁢nm-A620⁢nm)

Total anthocyanin content was normalized to the fresh weight (FW) of each sample.

### High-Pressure Liquid Chromatography Analysis

Accurately weighed leaf samples (0.5 g) were leached with 10 ml of methanol at 4°C for 48 h in the dark and then stirred by ultrasonic waves for 1 h. Mixtures were centrifuged at 6,000 rpm for 3 min at 4°C and then set aside. Materials were separated using a Shimadzu LC-2030C liquid chromatograph (Shimadzu, Kyoto, Japan) equipped with an Inertsil C-18 column (5.0 mm particle size, 4.6 mm × 250 mm). The HPLC separation was performed as previously described by Han et al. (2020a). The respective structure was confirmed by comparison with a standard using LC–MS as described by Li et al. (2007). Measured results were compared with retention time and standard curve of the reference substance. Three biological replicates were analyzed in this section.

### RNA Sequencing Data Analysis

Extraction of total RNA, library construction, RNA sequencing (RNA-Seq), RNA assembly, and DEG analysis of *M*. ‘Radiant’ leaves (S1 and S4, each with three biological replicates) were performed by Gene *Denovo* Biotechnology Co., (Guangzhou, China) as described previously (Han et al., 2020b). *Malus* × *domestica* HFTH1 Whole Genome version 1.0^1^ was used to conduct transcriptome alignment.

All DEGs were mapped to the Gene Ontology (GO) database^2^ and the Kyoto Encyclopedia of Genes and Genomes (KEGG) pathways database.^3^ Gene numbers were calculated for every term, and significantly enriched GO terms in DEGs, compared with the genome background, were defined by hypergeometric test with FDR ≤ 0.05 as a threshold.

### Quantitative Real-Time PCR Analysis

Total RNA was isolated following the method described previously. Approximately 1 μg of total RNA was used for first-strand cDNA synthesis using a PrimeScript™ RT reagent kit with a gDNA Eraser (TaKaRa Bio Inc., Shiga, Japan). Gene specific primers were designed with Primer Premier 5.0 (PREMIER Biosoft International, Palo Alto, CA, United States) and primer sequences are shown in Supplementary Table 1. The relative expression level of DEGs in the samples was determined using 2 × SYBR real-time PCR mixture kit (BioTeKe, Beijing, China) on the StepOnePlus real-time PCR system (Applied Biosystems, Waltham, MA, United States). 18S rRNA (DQ341382) was used as the internal control. All experiments were performed with three independent biological replicates and three technical replicates.

### Construction of Expression Vectors

To construct *MrMYB44-like1/2/3* overexpression vectors, *MrMYB44-like1/2/3* cDNA sequences without a termination codon were inserted between *Bam*HI and *Sal*I of pCAMBIA2300-green fluorescent protein (GFP) vectors (35S: GFP). In addition, a 152-bp fragment of *MrMYB44-like3* (411–563 bp), which is highly conserved in *MrMYB44-like1*, *MrMYB44-like2*, and *MrMYB44-like3*, was inserted between Kpn1 and Xhol of pTRV2 vectors to suppress *MrMYB44-like* gene expression. A Seamless Cloning and Assembly Kit (Novoprotein, Shanghai, China; primers are listed in Supplementary Table 1) was used to produce overexpression vectors 35S:*MrMYB44-like1*/2/3: GFP and gene silencing vector pTRV2-MrMYB44-like. Then, recombinant plasmids 35S:*MrMYB44-like1*/2/3: GFP, pTRV2-MrMYB44-like, pCAMBIA2300-35S: GFP empty vector, and pTRV1 and pTRV2 empty vectors were introduced into *Agrobacterium tumefaciens* GV3101 by the heat shock method.

### Subcellular Localization

The 35S:*MrMYB44-like1*/2/3: GFP vectors described in the last section were used to identify subcellular localization of *MrMYB44-like*s. Recombinant (35S:*MrMYB44-like1*/2/3: GFP) and control (pCAMBIA2300-35S: GFP) vectors were introduced into 5-week-old *Nicotiana benthamiana* leaves by agroinfiltration. Infiltrated plants were grown for over 72 h in a growth chamber, and the GFP fluorescence of samples was observed under a confocal laser-scanning microscope (TCS SP8; Leica, Wetzlar, Germany).

### Agroinfiltration in Fruit of *Malus domestica* ‘Fuji’

Procedures for overexpression and suppression of *MrMYB44-like*s by agroinfiltration in apple fruit were according to Li et al. (2012) with some modifications. *Agrobacterium tumefaciens* carrying pC2300-MrMYB44-like1/2/3 and empty pC2300 vectors were injected into the peel of freshly bagged apples using a needle-less syringe. Injected apples were treated in darkness at 4°C for 7 day and then transferred to 24 h of continuous white light (200 μmol m^–2^ s^–1^) with supplemental UV-B (280–320 nm) at 17°C in a growth chamber for 4 day.

To suppress *MrMYB44-like* expression, separate *A*. *tumefaciens* cultures containing pTRV1 and pTRV2-MrMYB44-like were mixed in a 1:1 ratio and then infiltrated into the fruit skin of freshly bagged apples using a needle-less syringe. Injected apples were treated as described above.

### Agroinfiltration in Leaf Disks of *Malus* ‘Radiant’

Overexpression, suppression, and empty vector injections were prepared as described previously. Agroinfiltration in leaf disks was performed according to procedures described by Dai et al. (2012) with some modifications. Leaves (S1) of *M.* ‘Radiant’ were collected from trees grown in natural conditions. One-centimeter-diameter disks were taken from the center of leaves with a hole punch. Leaf disks were placed in a bacterial suspension solution and infiltrated under vacuum at 0.5 MPa for 15 s. After release of the vacuum, disks were washed in deionized water twice and kept in deionized water for 3 day at 4°C and then at 24°C for 3 day.

### Bimolecular Fluorescence Complementation Assays

Constructs to investigate interactions were produced in pSPYNE-35S and pSPYCE-35S vectors by using bimolecular fluorescence complementation (BiFC) assays. The cDNAs without stop codons of *MrMYB44-like1/2/3* were cloned into pSPYNE-35S, and those of *MrbHLH3* and *MrWRKY6* were cloned into pSPYCE-35S. Primers used for plasmid construction are listed in Supplementary Table 1. Constructs were transformed into *A. tumefaciens* by the heat shock method. Five-week-old *Nicotiana benthamiana* leaves were infiltrated with the mixed *A. tumefaciens*. Fluorescence signals were detected 72 h after infiltration using an inverted laser-scanning microscope with a 40 × water-immersion objective (TCS SP8; Leica, Wetzlar, Germany).

## Results

### Analysis of Pigment Levels and Differentially Expressed Genes in Spring Leaves

Leaves of *M.* ‘Radiant’ changed from red to green during spring growth (Figure 1A). The values of chromatic parameters L* and b* increased gradually and peaked in S4 (Figure 1B). By contrast, values of a* decreased to negative in S3 and reached the lowest value in S4. To investigate physiological changes in leaves of different colors, total anthocyanin content was determined by extraction with methanol–HCL. Total anthocyanin content was highest in S1, reaching 4.79 μmol g^–1^ FW, and then decreased gradually in other stages, reaching 0.43 μmol g^–1^ FW in S4 (Figure 1C). Anthocyanin constituents and contents in the four leaf stages were determined using HPLC. Cyanidin 3-galactoside chloride and cyanidin-3-O-glucoside chloride were both found in S1 and S2, but cyanidin-3-O-glucoside chloride was not detected in S3 and S4. Changes in contents of the two compounds were consistent with those of total anthocyanin (Table 1). Nine other types of phenolic compounds were also identified, including five flavonols, two flavanols, and one flavone (Table 1). Because anthocyanin contents were significantly different (*P* < 0.05), stages S1 and S4 were selected for RNA-sequencing.

**TABLE 1:** The HPLC results of leaf extraction of *Malus* ‘Radiant.’

Classification	Polyphenol (mg/g)	S1	S2	S3	S4
Anthocyanin	Cyanidin-3-galactoside chloride	0.658 ± 0.051^a^	0.370 ± 0.041^b^	0.287 ± 0.037^c^	0.093 ± 0.007^d^
	Cyanidin-3,5-O-diglucoside	0.093 ± 0.023^a^	0.022 ± 0.006^b^	ND	ND
Flavonol	Hyperoside	1.572 ± 0.136^a^	1.638 ± 0.110^a^	1.167 ± 0.077^b^	0.902 ± 0.073^c^
	Rutin	0.072 ± 0.017^a^	0.008 ± 0.005^b^	0.007 ± 0.005^b^	ND
Flavanol	Catechin	0.123 ± 0.019^b^	0.152 ± 0.012^ab^	0.172 ± 0.009^a^	0.159 ± 0.022^a^
	Epicatechin	0.049 ± 0.009^ab^	0.078 ± 0.014^a^	0.085 ± 0.039^a^	0.029 ± 0.018^b^
	Procyanidins B1	0.129 ± 0.007^a^	0.131 ± 0.005^a^	0.123 ± 0.001^a^	0.111 ± 0.003^b^
	Procyanidins B2	0.002 ± 0.0003^c^	0.022 ± 0.001^a^	0.011 ± 0.001^b^	ND
	Chlorogenic acid	0.544 ± 0.011^a^	0.510 ± 0.009^b^	0.497 ± 0.028^b^	0.491 ± 0.014^b^
Flavone	Naringenin	0.154 ± 0.015^c^	0.214 ± 0.046^c^	0.311 ± 0.047^b^	0.475 ± 0.062^a^

*The results are presented as mean ± SD. Different letters between cultivars denote significant differences (Duncan test, p < 0.05).*

Six RNA libraries from *M*. ‘Radiant’ leaves (S1 and S4, each with three biological replicates) were sequenced. After removing adapter sequences and low-quality reads, raw reads of each sample were equal to or greater than 48,141,832 (Supplementary Table 2). Clean reads with a Q-score of 30 or higher (i.e., base call accuracy ≥ 99.9%) accounted for more than 94% of the total. The GC content (ratio of guanine and cytosine content to total nucleobases) ranged from 47 to 49%. Total mapping of each library was greater than 89%, and average unique mapping was greater than 85%. These results indicated transcriptomic data were suitable for further analysis.

**FIGURE 2:**
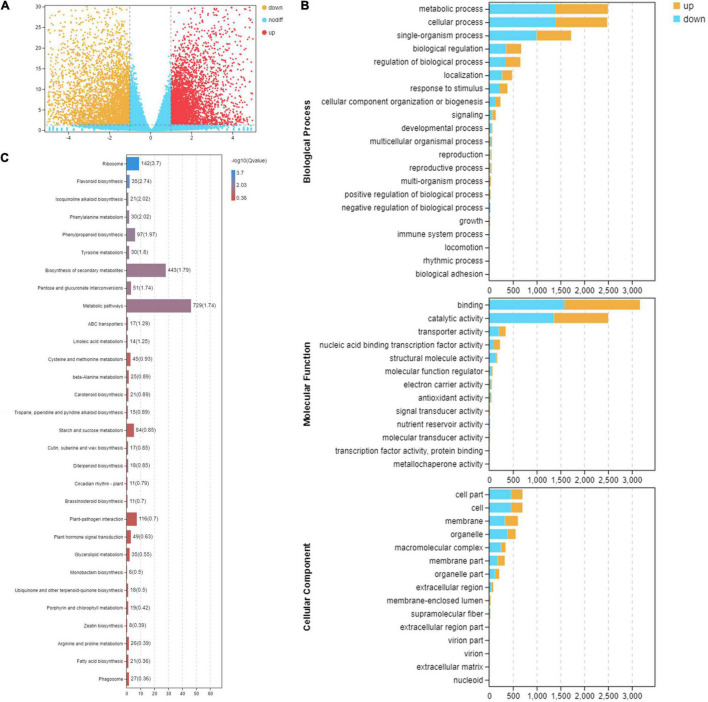
Functional analysis of differentially expressed genes between consecutive developmental stages. (**A**) The volcano distribution map of differentially expressed genes in the leaves at stage 1 (S1) and stage 4 (S4) on RNA-seq experiments. The genes were classified into three classes. Yellow genes are up-regulated gene expression, red indicates up-regulated gene expression, and blue indicates genes were not differentially expressed. The *x*-axis represents log_2_(fold change); the *y*-axis represents -log10 values. **(B)** Gene Ontology enrichment analysis in pairwise comparison between S1 and S4. Yellow indicates up-regulated genes, and blue indicates down-regulated genes. **(C)** Kyoto Encyclopedia of Genes and Genomes pathway enrichment analysis in pairwise comparison between S1 and S4.

Differentially expressed genes between the two leaf stages were identified. The overall distribution of DEGs is shown in a volcano plot (Figure 2A). A total of 14,660 DEGs were detected (Figure 2A). In the comparison between S1 and S4, 8,790 DEGs were identified, with 4,071 genes up-regulated and 4,719 genes down-regulated (Figure 2A).

### Gene Ontology and Kyoto Encyclopedia of Genes and Genomes Pathway Enrichment Analyses of Differentially Expressed Genes

In the GO classification, 14,660 matched DEGs were categorized into 49 GO terms in three major GO ontologies, with 21 in biological process, 13 in molecular function, and 15 in cellular component (Figure 2B). In the KEGG database, 1,503 DEGs were mapped to 130 pathways, representing metabolism, genetic information processing, organism systems, cellular processes, and environmental information processing. Moreover, pathways closely associated with change in leaf pigmentation were significantly enriched, including flavonoid biosynthesis, phenylalanine metabolism, phenylpropanoid biosynthesis, biosynthesis of secondary metabolites, carotenoid biosynthesis, and porphyrin and chlorophyll metabolism (Figure 2C). The genes involved in pathways related to change in leaf pigmentation in *M*. ‘Radiant’ were investigated further.

### Analysis of Genes Associated With Anthocyanin Metabolites

**FIGURE 3:**
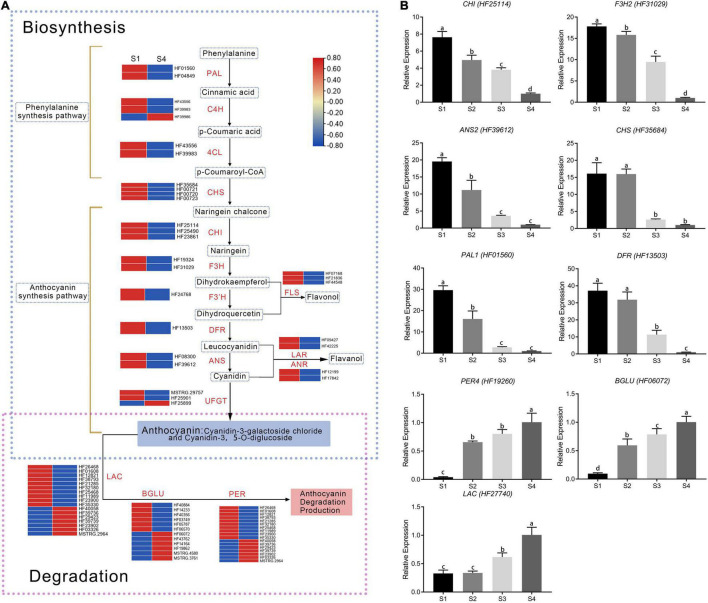
Expression of differentially expressed genes involved in anthocyanin pathways. **(A)** Expression analysis of flavonoid pathway genes at S1 and S4 stages in fruit. Expression was evaluated *via* RNA-seq with three biological replicates. Log_2_ fold change represents expression. **(B)** Validation of RNA-seq expression profiles in stages S1–S4 *via* real-time quantitative RT-PCR. Values were calculated relative to the transcription level in S4 (set to one). Error bars represent the standard errors. Different lowercase letters indicate significant differences among treatments according to one-way ANOVA test (*P* < 0.05).

Twenty-two DEGs were involved in anthocyanin biosynthesis (Figure 3A). Phenylpropanoid and polyketide pathways contained two *PAL*, three *C4H*, and two *4CL* genes. Early anthocyanin biosynthesis involved four *CHS*, three *CHI*, two *F3H*, and one *F3*’*H* gene, whereas one *DFR*, two *ANS*, and three *UFGT* genes were involved in later stages of biosynthesis. Most enzyme-encoding genes had higher FPKM (fragment per kilobase of transcript per million mapped reads) values in S1 than in other stages, except one *C4H* and one *UFGT* (Figure 3A). Consistent with low anthocyanin content in S4, expression of most genes was also very low (Supplementary Table 3).

Genes with FPKM values less than one were excluded in the study. The remaining targets included 18 laccase genes, 17 peroxidase genes, and 12 β-glucosidase genes (Figure 3A). Among them, 10 laccase, 10 peroxidase, and 6 β-glucosidase genes had relatively low expression in S1 but relatively high expression in S4. Those genes were negatively correlated with anthocyanin content (Supplementary Table 3), indicating they promoted anthocyanin degradation.

Results from quantitative real-time PCR (qRT-PCR) were used to validate whether differences in RNA-seq levels of DEGs involved in the anthocyanin metabolism pathway reflected actual transcription levels in *M*. ‘Radiant’ leaves at different stages of development (Figure 3B). Expression patterns of six anthocyanin biosynthesis genes [*MrPAL* (HF01560), *MrCHS* (HF35684), *MrCHI* (HF25114), *MrF3H* (HF31029), *MrDFR* (HF13503), and *MrANS* (HF39612)] and three anthocyanin degradation genes [*MrLAC* (HF27740), *MrPER4* (HF19260), and *MrBGLU* (HF06072)] obtained by qRT-PCR were consistent with RNA-Seq data, confirming expression patterns in the four stages of development (Figure 3B). Expression levels of anthocyanin biosynthesis genes gradually decreased as red faded from leaves. By contrast, the anthocyanin degradation genes *MrLAC*, *MrPER4* and *MrBGLU* were most highly expressed in S4.

To screen potential regulators of anthocyanin biosynthesis, 12 TF families were identified. After removal of genes with extremely low expression (FPKM < 1), 44 MYBs, 37 bHLHs, 1 WD40, 34 WRKYs, 8 SPLs, 2 HY5s, 7 LBDs, 22 HSPs, 11 T, 38 ERFs, 94 NACs, and 4 bZIPs were identified. In MYB, WRKY, HSP, ERF and bZIP families, more genes had higher expression in S4 than in S1. By contrast, in bHLH, WD40, SPL, HY5, LBD, TCP, and NAC families, more genes had lower expression in S4 than in S1 (Table 2). All TF families had complex expression patterns (Supplementary Table 4). Gene expression in the same family was either positively or negatively correlated with anthocyanin content, which indicated a complex network regulated change in leaf coloration.

**TABLE 2:** Candidate anthocyanin regulatory genes in *Malus* ‘Radiant.’

Gene family	NO. all^a^	S1 vs. S4	
		NO. up^b^	NO. down^c^
MYB	44	24	20
bHLH	37	12	25
WD40	1	0	1
WRKY	34	30	4
SPL	8	1	7
HY5	2	0	2
LBD	7	1	6
HSP	22	13	9
TCP	11	5	6
ERF	38	23	15
NAC	94	38	56
bZIP	4	4	0

*^a^Indicates the total number of regulatory genes in differentially expressed genes.*

*^b^Indicates the number of upregulated genes in each comparison.*

*^c^Indicates the number of downregulated genes in each comparison. All candidate anthocyanin regulatory genes in this table are screened from differentially expressed genes.*

### Analysis of MYB Transcription Factors Identified as Differentially Expressed Genes

**FIGURE 4:**
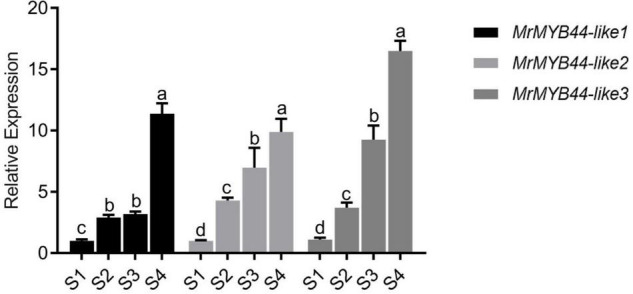
Transcript levels of *MrMYB44-like1*, *MrMYB44-like2*, and *MrMYB44-like3* at different stages of development (S1–S4) in *Malus* ‘Radiant.’ Values were calculated relative to the transcription level of S1 (set to one). Error bars represent the standard errors. Different lowercase letters indicate significant differences among treatments according to one-way ANOVA test (*P* < 0.05).

In RNA-Seq data, 44 MYBs were identified as DEGs (both R2R3 MYBs and R3 MYBs). Heat map and motif analysis are shown in Supplementary Figure 1. Notably, expression of three MYBs (HF13071, HF21590, and HF27377) increased 2.76 to 3.68-log_2_fold in S4, with FPKM values ranging from 91.07 to 257.49, which were higher than those of most of other MYB DEGs (Supplementary Table 3). Results from qRT-PCR showed that expression of all three genes increased as leaves changed from red to green in S1–S4, consistent with RNA-seq expression patterns (Figure 4). These results suggested the three MYBs negatively regulated anthocyanin biosynthesis.

Phylogenetic analysis was performed on the differentially expressed MYB TFs and 126 *Arabidopsis* MYBs from the TAIR^4^ (Supplementary Figure 2). The 44 MYB DEGs were divided into 24 subgroups. The three MYBs (HF13071, HF21590, and HF27377) were in subgroup 22 which including *AtMYB44* (AT5G67300), *AtMYB70* (AT2G23290), *AtMYB73* (AT4G37260), and *AtMYB77* (AT3G50060) (Supplementary Figure 2). Then, all of these three MYBs were used for basic local alignment search tool (BLAST)p queries against the genome of *Malus* in National Center for Biotechnology Information (NCBI) GenBank database, and they were close BLAST match to three *MdMYB44-like* genes, respectively. HF13071 is 100% match to NP_001315844.1, and HF21590 is 100% match to NP_001287808.1, and HF27377 is 99.68% match to XP_028963833.1. Therefore, HF13071, HF21590, and HF27377 were designated as *MrMYB44-like1*, *MrMYB44-like2*, and *MrMYB44-like3*, respectively. In addition, the 44 MYBs were located on chromosomes 1–17 (Supplementary Figure 3).

### Cloning and Subcellular Localization of MrMYB44-Likes

Full-length sequences of *MrMYB44-like1*, *MrMYB44-like2*, and *MrMYB44-like3* were obtained from RNA-seq, and cDNA prepared from *M*. ‘Radiant’ S4 leaf RNA was used as templates for PCR amplification of gene sequences (Supplementary Table 1). Amino acid sequence alignment indicated the deduced proteins of *MrMYB44-like1*, *MrMYB44-like2*, and *MrMYB44-like3* shared 41.1, 39.6, and 48.45% sequence identity, respectively, with *AtMYB44*. As shown in Supplementary Figure 4, *MrMYB44-like1*, *MrMYB44-like2*, and *MrMYB44-like3* all contained the conserved motifs of subgroup 22, including 22.1 (TGLYMSPxSP), 22.2 (D/EPP/MTxLxLSLP), and 22.3 (GxFMxVVQEMIxxEVRSYM) (Stracke et al., 2010; Zhou et al., 2017). Motif 22.2 is partially conserved with the EAR motif found in subgroup 4 R2R3 MYB repressors. Additionally, the conserved bHLH-interacting motif amino acid ([D/E]Lx2[R/K]x3Lx6Lx3R) was not found in *MrMYB44-like*s and other amino acid sequences except in *AtMYB4* and *FaMYB1*. In addition, genes *MrMYB44-like1*, *MrMYB44-like2*, and *MrMYB44-like3* were on chromosomes 15, 8, and 2, respectively (Supplementary Figure 3).

To examine subcellular localization of *MrMYB44-like1/2/3*, recombinant (35S:*MrMYB44-like1/2/3*: GFP) and control (pCAMBIA2300-35S: GFP) vectors were introduced into tobacco leaves by agroinfiltration. The GFP fluorescence of the control vector was clearly distributed throughout the entire cell (Supplementary Figure 4A), whereas 35S:*MrMYB44-like1/2/3*: GFP vectors displayed strong fluorescence signals in the nuclei of tobacco cells (Supplementary Figures 4B–D). Therefore, the three R2R3-MYB TFs, *MrMYB44-like1/2/3*, were likely localized and functioned in the nucleus.

### MrMYB44-Like Negatively Regulates Peel Coloration in *Malus domestica* ‘Fuji’

To verify *MrMYB44-like*s down-regulated anthocyanin biosynthesis, overexpression vectors (pCambia2300) of *MrMYB44-like1*, *MrMYB44-like2*, and *MrMYB44-like3* were constructed. Overexpression was initiated by an agrobacterium-mediated transformation method. Gene expression was suppressed by virus-induced gene silencing (VIGS), using a TRV vector. Vectors contained a conserved *MrMYB44-like* region, and in principle, could silence the expression of *MrMYB44-like1*, *MrMYB44-like2*, and *MrMYB44-like3*.

**FIGURE 5:**
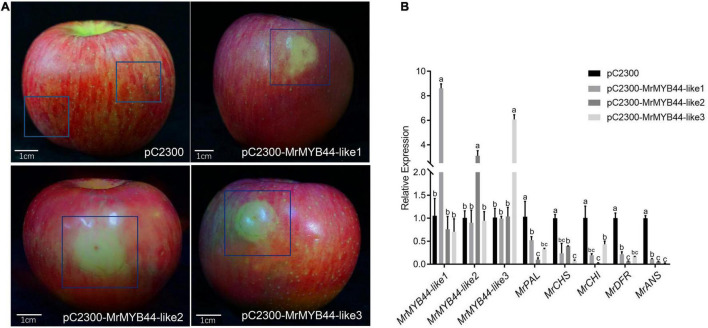
*MrMYB44-like*s negatively regulate color formation in apple transformation assays. **(A)** *Malus domestica* ‘Fuji’ fruit peel coloration around injection sites. pC2300-MYB44-likes were used for overexpression with the pCambia2300 vector. Empty vectors were the control. **(B)** Transcript levels of *MrMYB44-like*s and other anthocyanin biosynthesis genes in *M*. *domestica* ‘Fuji’ fruit peel around injection sites. Values were calculated relative to the transcription level of the control (set to one). Error bars represent the standard errors. Different lowercase letters indicate significant differences among treatments according to one-way ANOVA test (*P* < 0.05).

**FIGURE 6:**
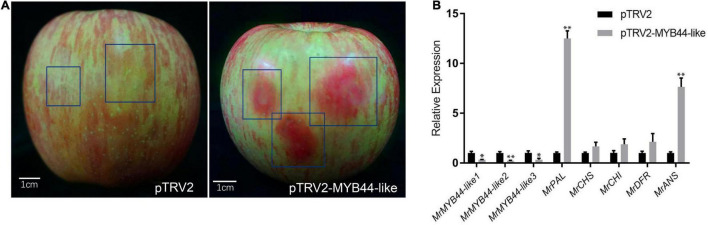
*MrMYB44-like*s negatively regulate color formation in apple in viral vector-based transformation assays. **(A)** *Malus domestica* ‘Fuji’ fruit peel coloration around injection sites. Antisense *MrMYB44-like* (pTRV2-MrMYB44-like) was used for suppression with the TRV vector. Empty TRV2 vectors were the control. **(B)** Transcript levels of *MrMYB44-like*s and other biosynthesis genes in *M*. *domestica* ‘Fuji’ fruit peel around injection sites. Error bars represent the standard errors. *P < 0.05, ^**^P < 0.01 using the *t*-test.

Overexpression of *MrMYB44-like1*, *MrMYB44-like2*, and *MrMYB44-like3* inhibited fruit peel coloration around injection sites, whereas there was obvious red coloration in sites without injection and with injection of empty pCambia2300 vectors (Figure 5A). Subsequently, RT-qPCR revealed that *MrMYB44-like1*, *MrMYB44-like2*, and *MrMYB44-like3* transcript levels in overexpression peel sites increased by approximately three- to six-fold (Figure 5B), whereas expression of anthocyanin biosynthesis genes, such as *MrPAL*, *MrCHS*, *MrCHI*, *MrDFR*, and *MrANS*, decreased (Figure 5B). By contrast, with silencing of *MrMYB44-like*s, red pigmentation increased around injection sites, compared with sites without injection and with injection of empty TRV2 vectors (Figure 6A). Expression levels of the three *MrMYB44-like*s decreased by approximately three- to four-fold, whereas those of the anthocyanin biosynthesis genes greatly increased by approximately two- to twelve-fold (Figure 6B). The abundance of those gene transcripts was consistent with pigmentation.

### MrMYB44-Like Negatively Regulates Anthocyanin Biosynthesis in Leaf Disks of *Malus* ‘Radiant’

**FIGURE 7:**
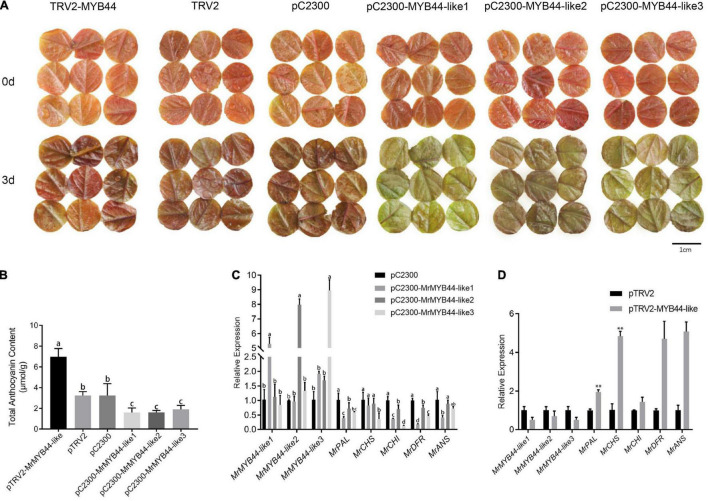
Relative anthocyanin contents and transcript levels of genes in the anthocyanin biosynthesis pathway in *MrMYB44-like*-overpression/repression transgenic leaf disks and empty vector control leaf disks of *Malus* ‘Radiant.’ **(A)** The phenotype of transgenic and control disks. **(B)** Relative anthocyanin content of transgenic and control disks. **(C)** Transcript levels of *MrMYB44-like*s and structural genes related to anthocyanin biosynthesis in *MrMYB44-like*-overexpression and control leaf disks. Error bars represent the standard errors. Different lowercase letters indicate significant differences among treatments according to one-way ANOVA test (*P* < 0.05). **(D)** Transcript levels of *MrMYB44-like*s and structural genes related to anthocyanin biosynthesis in *MrMYB44-like*-silenced and control leaf disks. Error bars represent the standard errors. *P < 0.05, ^**^P < 0.01 using the *t*-test.

To verify *MrMYB44-like*s repressed anthocyanin biosynthesis in crabapple leaves, red, young leaf disks collected in spring were infected with overexpression and suppression recombinant vectors. There was inevitable passive anthocyanin degradation because the leaves were cultured *in vitro*, and as a result, empty vector (both pCambia2300 and pTRV2)-infected leaf disks also faded to some degree at 3 days post-infection. However, fading of red in the overexpression group was greater than that in those untreated and treated with empty vectors as leaves turned green, whereas the silenced group maintained the red phenotype (Figure 7A).

Total anthocyanin concentration in overexpression leaf disks (1.60, 1.61, and 1.92 μmol g^–1^ in pC2300-MYB44-like1, pC2300-MYB44-like2, and pC2300-MYB44-like3, respectively) was approximately twofold lower than that in leaf disks with empty pC2300 vectors (3.25 μmol g^–1^). In addition, anthocyanin concentration in silenced leaf disks (6.98 μmol g^–1^, with pTRV2-MYB44-like) was approximately twofold higher than that in leaf disks of the control group (3.25 μmol g^–1^, with pTRV2) (Figure 7B). According to HPLC analyses, concentrations of both cyanidin-3-galactoside chloride and cyanidin-3,5-O-diglucoside with overexpression or suppression of *MrMYB44-like*s were approximately twofold lower or higher, respectively, than the concentrations in the corresponding control groups. Those results were consistent with total anthocyanin concentrations and phenotype coloration (Supplementary Table 5). Moreover, levels of rutin, hyperoside, and naringenin in silenced leaf disks were higher than those in control leaves, whereas with overexpression, levels were lower than those in control leaves.

According to qRT-PCR results, expression of *MrMYB44-like1*, *MrMYB44-like2*, and *MrMYB44-like3* increased by five-, eight-, and nine-fold, respectively, in overexpression leaf disks. By contrast, expression of anthocyanin biosynthesis genes, including *MrPAL*, *MrCHS*, *MrCHI*, *MrDFR*, and *MrANS*, decreased significantly (Figure 7C). Furthermore, *MrMYB44-like1*, *MrMYB44-like2*, and *MrMYB44-like3* transcript levels in *MrMYB44-like*-silenced leaf disks decreased by approximately 50%, compared with levels in leaf disks with empty vectors. Transcript levels of anthocyanin biosynthesis genes were significantly higher in silenced leaf disks than in control leaf disks (Figure 7D). Overall, expression of these genes was consistent with anthocyanin levels and the pigmentation observed in overexpression, silenced, and control groups.

### MrMYB44-Likes Interact With MrWRKY6 but Not With MrbHLH3

**FIGURE 8:**
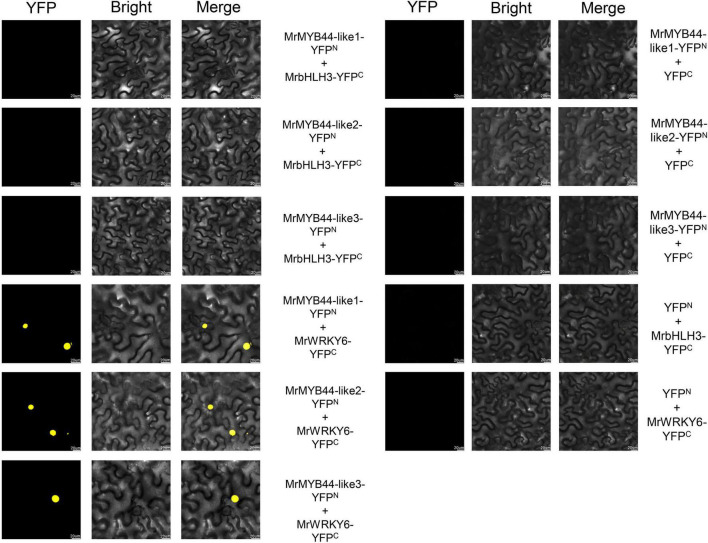
Bimolecular fluorescence complementation assays showing that *MrMYB44-like1/2/3* did not interact with *MrbHLH3* but did interact with *MrWRKY6* in nuclei of epidermal cells in *Nicotiana benthamiana*. Bars, 20 μm.

According to the previous studies, combinatorial interactions between MYB and bHLH TFs are crucial in regulating anthocyanin biosynthesis (Albert et al., 2011; Xu et al., 2018). However, the three *MrMYB44-like*s had no bHLH-interacting sites according to amino acid sequence alignment. To further investigate whether there were interactions between *MrMYB44-like*s and *MrbHLH*, BiFC assays were performed. The gene *MrbHLH3* (HF28271) was screened because of its close genetic relationship with *MdbHLH3*, which was previously characterized as a regulator of the flavonoid pathway in *Malus* (Xie et al., 2012). Three construct combinations, that is, MrMYB44-like1-YFP*^N^* plus MrbHLH3-YFP*^C^*, MrMYB44-like2-YFP*^N^* plus MrbHLH3-YFP*^C^*, and MrMYB44-like3-YFP*^N^* plus MrbHLH3-YFP*^C^*, were cotransformed into tobacco epidermal cells. Yellow fluorescent signals were not observed in epidermis cells transformed with any of the three combinations (Figure 8). The results suggested that *MrMYB44-like1/2/3* did not interact with *MrbHLH3* to suppress anthocyanin biosynthesis.

According to Zhou et al. (2017), *StMYB44*, in MYB subgroup 22, physically interacts with *AtWRKY6* and *StWRKY6 in vivo*. Therefore, BiFCs were performed to investigate whether *MrWRKY6* could interact with *MrMYB44-like1/2/3* to form protein complexes. *MrWRKY6* was fused to the C-terminal fragment of YFP as MrWRKY6-YFP*^C^*, and then, three combinations including MrMYB44-like1-YFP*^N^* plus MrWRKY6-YFP*^C^*, MrMYB44-like2-YFP*^N^* plus MrWRKY6-YFP*^C^*, and MrMYB44-like3-YFP*^N^* plus MrWRKY6-YFP*^C^* were cotransformed into tobacco epidermal cells. As shown in Figure 8, yellow fluorescence signals were observed in nuclei of tobacco cells that coexpressed one of the three groups. Thus, MrWRKY6 physically interacted with *MrMYB44-like1/2/3* in the nucleus. By contrast, there was no signal with the combinations of MrMYB44-like1-YFP*^N^* plus YFP*^C^*, MrMYB44-like2-YFP*^N^* plus YFP*^C^*, MrMYB44-like3-YFP*^N^* plus YFP*^C^*, and YFP*^N^* plus MrWRKY6-YFP*^C^*. The results demonstrated that *MrWRKY6* physically interacted with *MrMYB44-like1/2/3 in vivo*.

## Discussion

In spring, leaf color changes from red to green during growth in *M*. ‘Radiant.’ The value of a*, which is a standard for red and green, gradually decreased from S1 to S4. Consistent with color measurements, HPLC results showed contents of cyanidin 3-galactoside chloride and cyanidin-3-O-glucoside chloride decreased with development. Therefore, decreasing anthocyanin content led to fading red color in *M*. ‘Radiant’ leaves.

The MYB TFs positively or negatively regulate anthocyanin biosynthesis (Yan et al., 2021). In this study, 44 MYBs, including 38 R2R3-MYBs and 6 R3-MYBs, were screened in a phylogenetic tree analysis, which indicated that R2R3-MYBs have crucial roles as transcriptional regulators affecting changes in leaf color. Among the MYBs, *MrMYB44-like1* (HF13071), *MrMYB44-like2* (HF21590), and *MrMYB44-like3* (HF27377) had notably high FPKM and fold change values, and expression of the three genes was negatively correlated with anthocyanin content. *MrMYB44-like1/2/3* are in subgroup 22, which has a partial EAR motif (Liu et al., 2019). EAR motif-mediated transcriptional repression is the main form of transcriptional repression in plants (Jiao et al., 2018). Most importantly, transient overexpression of *MrMYB44-like1*, *MrMYB44-like2*, and *MrMYB44-like3* in *M*. *domestica* ‘Fuji’ fruit peels and *M*. ‘Radiant’ leaves repressed expression of structural genes related to anthocyanin biosynthesis and reduced anthocyanin concentrations. By contrast, structural gene expression and red pigmentation increased significantly when *MrMYB44-like*s were transiently silenced in peels and leaves. Therefore, *MrMYB44-like*s are likely functional MYB TFs that negatively regulate anthocyanin biosynthesis. The three *MYB44-like* genes all showed strong inhibition ability when transiently overexpressed, although their amino acid sequences were not identical. The genes were also on different chromosomes. Those results indicated they were functionally redundant genes in the same subgroup of MYBs rather than different copies of one gene or allele genes. The three gene sequences were similar to *MdMYB44* which was reported to negatively regulate fruit malate accumulation (Jia et al., 2018, 2021). It was indicated that the *MYB44* or *MYB44-like* may play as important repressors in secondary metabolism in *Malus*.

In plants, R2R3-MYB repressors can passively compete with activator complexes by interacting with bHLH proteins to reduce their activation capability and thereby repress anthocyanin biosynthesis (Albert et al., 2011; Chen L. et al., 2019; Yan et al., 2021). However, in this study, according to amino acid sequence alignment, *MrMYB44-like1/2/3* did not contain the conserved amino acid signature of the bHLH-interacting motif ([D/E]Lx2[R/K]x3Lx6Lx3R), which is used to predict new MYB–bHLH interactions (Zimmermann et al., 2010). Further, in BiFC assays, *MrMYB44-like*s did not interact with *MrbHLH3*, indicating the repressive function is independent of bHLH. Thus, *MrMYB44-like*s could not repress anthocyanin biosynthesis by interacting with bHLH proteins to compete with MYB activators in the MYB–bHLH complex.

In addition to bHLH, many other TFs interact with MYBs, such as *AtWRKY6* and *StWRKY6*, which physically interact with *StMYB44 in vivo* (Zhou et al., 2017). Amino acid sequence alignment indicated *MrMYB44-like1/2/3* shared high amino acid identity with *StMYB44* in DNA-binding domains. In addition, *MrWRKY6* (HF12290) had affinities with *AtWRKY6* and *StWRKY6*, and according to RNA-seq, expression of *MrWRKY6* was consistent with that of *MrMYB44-like1/2/3*. Therefore, interactions were predicted between *MrWRKY6* and *MrMYB44-like1/2/3*, and BiFC assays confirmed that *MrMYB44-like1/2/3* physically interacted with *MrWRKY6* in nuclei of tobacco epidermal cells. Those results suggested that *MrMYB44-like1*, *MrMYB44-like2*, and *MrMYB44-like3* could form a protein complex with *MrWRKY6* to regulate transcript levels of anthocyanin biosynthesis genes. In mature leaves, high abundance of transcriptional complexes likely strongly inhibited anthocyanin biosynthesis genes, which led to fading color. In previous studies, *WRKY6* responded to plant senescence, in pathogen defense, and to low phosphorus stress in different plants (Robatzek and Somssich, 2002; Wang et al., 2016; Zhou et al., 2017; Chen Z. et al., 2019). In this study, a different potential function of *WRKY6* was identified that indicated it might be a key regulator of the anthocyanin biosynthesis pathway. However, further studies are needed.

## Conclusion

This study confirms that in spring, anthocyanin content decreases with leaf development in *M*. ‘Radiant.’ During development, transcription of most anthocyanin biosynthesis genes gradually decreases and that of key anthocyanin degradation genes gradually increases. The MYB repressors *MrMYB44-like1*, *MrMYB44-like2*, and *MrMYB44-like3* are members of subgroup 22 and are important negative regulators that inhibit anthocyanin biosynthesis. The three *MrMYB44-like*s interact with *MrWRKY6* but not with *MrbHLH3*. This study further validates and supplements known functions of MYB subgroup 22, while also providing new insights into the mechanism of leaf color change in crabapple.

## Data Availability Statement

The datasets generated for this study can be found in the NCBI Sequence Read Archive (SRA) with bioproject No. PRJNA784337, and under GenBank accession numbers of SAMN23483827, SAMN23483828, SAMN23483829, SAMN23483830, SAMN23483831, and SAMN23483832.

## Author Contributions

J-XM performed most of the experiments and data analysis. JW and R-FC carried out material collection and pigment extraction. Y-HQ, R-FC, and HW conducted pigment analysis. JZ conducted a part of RNA extraction and qRT-PCR experiment. JW and Y-LW constructed a part of vectors. J-XM, JW, Y-HQ, and R-FC participated in the preparation of the manuscript. H-HL conceived, designed, and coordinated the studies. All authors contributed to the article and approved the submitted version.

## Conflict of Interest

The authors declare that the research was conducted in the absence of any commercial or financial relationships that could be construed as a potential conflict of interest.

## Publisher’s Note

All claims expressed in this article are solely those of the authors and do not necessarily represent those of their affiliated organizations, or those of the publisher, the editors and the reviewers. Any product that may be evaluated in this article, or claim that may be made by its manufacturer, is not guaranteed or endorsed by the publisher.

## Footnotes

1
https://www.rosaceae.org/species/malus_x_domestica_HFTH1/genome_v1.0

2
http://www.geneontology.org/

3
http://www.kegg.jp/kegg

4
https://www.arabidopsis.org/
