# Supplementary material for: MrMYB44-Like Negatively Regulates Anthocyanin Biosynthesis and Causes Spring Leaf Color of Malus ‘Radiant’ to Fade From Red to Green
Source: Front Plant Sci. 2022 Feb 1;13:822340. doi: 10.3389/fpls.2022.822340 (PMC8843855; doi:10.3389/fpls.2022.822340)
Supplement: Supplementary file 2 [file Table_2.docx]

| **Sample** | **Raw Reads** | **Clean Reads** | **Clean Reads Ratio (%)** | **Clean Reads**  **Q30 (%)** | **Clean Reads**  **GC (%)** | **Total**  **Mapped (%)** | **Unique**  **Mapped (%)** |
| --- | --- | --- | --- | --- | --- | --- | --- |
| S1-1 | 54086648 | 54015674 | 99.87 | 94.08 | 48.52 | 90.84 | 87.87 |
| S1-2 | 57591832 | 57521216 | 99.88 | 94.97 | 48.55 | 91.30 | 87.96 |
| S1-3 | 53477834 | 53414346 | 99.88 | 94.71 | 48.64 | 91.76 | 88.70 |
| S4-1 | 48212192 | 48141832 | 99.85 | 95.08 | 48.22 | 90.09 | 86.37 |
| S4-2 | 54914960 | 54834648 | 99.85 | 94.61 | 47.97 | 90.62 | 86.67 |
| S4-3 | 55859546 | 55763442 | 99.83 | 94.81 | 47.87 | 89.67 | 85.56 |

**Supplementary Table 2.** Overall sequencing data
