# Supplementary material for: MrMYB44-Like Negatively Regulates Anthocyanin Biosynthesis and Causes Spring Leaf Color of Malus ‘Radiant’ to Fade From Red to Green
Source: Front Plant Sci. 2022 Feb 1;13:822340. doi: 10.3389/fpls.2022.822340 (PMC8843855; doi:10.3389/fpls.2022.822340)
Supplement: Supplementary file 5 [file Table_5.docx]

| Classification | Polyphenol (mg/g) | TRV2-MrMYB73s | TRV2 | PC2300 | PC2300-MrMYB73-1 | PC2300-MrMYB73-2 | PC2300-MrMYB73-3 |
| --- | --- | --- | --- | --- | --- | --- | --- |
| Anthocyanin | Cyanidin-3-galactoside chloride | 0.487±0.084^a^ | 0.256±0.060^b^ | 0.218±0.006^bc^ | 0.160±0.028^c^ | 0.133±0.012^c^ | 0.133±0.016^c^ |
|  | Cyanidin-3,5-O-diglucoside | 0.219±0.012^a^ | 0.114±0.005^b^ | 0.115±0.011^b^ | 0.069±0.004^c^ | 0.058±0.009^c^ | 0.066±0.006^c^ |
| Flavonol | Hyperoside | 0.421±0.016^a^ | 0.367±0.037^b^ | 0.354±0.008^b^ | 0.340±0.011^b^ | 0.347±0.035^b^ | 0.341±0.029^b^ |
|  | Lutin | 0.070±0.010^a^ | 0.038±0.020^b^ | 0.026±0.009^b^ | 0.022±0.002^b^ | 0.031±0.014^b^ | 0.023±0.009^b^ |
| Flavanol | Catechin | 0.120±0.055^a^ | 0.070±0.043^ab^ | 0.020±0.000^b^ | 0.034±0.018^b^ | 0.055±0.037^ab^ | 0.082±0.036^ab^ |
|  | Epicatechin | 0.059±0.028^a^ | 0.037±0.026^ab^ | 0.036±0.003^ab^ | 0.023±0.014^b^ | 0.007±0.005^b^ | 0.022±0.007^b^ |
|  | Procyanidins B1 | 0.169±0.009^b^ | 0.169±0.013^b^ | 0.163±0.002^b^ | 0.190±0.013^a^ | 0.150±0.012^b^ | 0.167±0.006^b^ |
|  | Procyanidins B2 | 0.024±0.004^a^ | 0.023±0.004^ab^ | 0.022±0.003^ab^ | 0.019±0.002^bc^ | 0.012±0.001^d^ | 0.014±0.001^cd^ |
|  | Chlorogenic acid | 0.507±0.006^a^ | 0.487±0.025^ab^ | 0.485±0.012^ab^ | 0.480±0.006^b^ | 0.486±0.011^ab^ | 0.485±0.006^ab^ |
| Flavone | Naringenin | 0.858±0.070^a^ | 0.361±0.042^bc^ | 0.423±0.174^b^ | 0.310±0.002^bc^ | 0.222±0.038^cd^ | 0.120±0.021^d^ |

**Supplementary Table 5.** **The HPLC results of leaf discs extraction in *Malus* ‘Radiant’.**

The results are presented as mean ± SD. Different letters between cultivars denote significant differences (Duncan test, *p* < 0.05).
