# Supplementary material for: MrMYB44-Like Negatively Regulates Anthocyanin Biosynthesis and Causes Spring Leaf Color of Malus ‘Radiant’ to Fade From Red to Green
Source: Front Plant Sci. 2022 Feb 1;13:822340. doi: 10.3389/fpls.2022.822340 (PMC8843855; doi:10.3389/fpls.2022.822340)
Supplement: Supplementary file 6 [file Data_Sheet_1.docx]

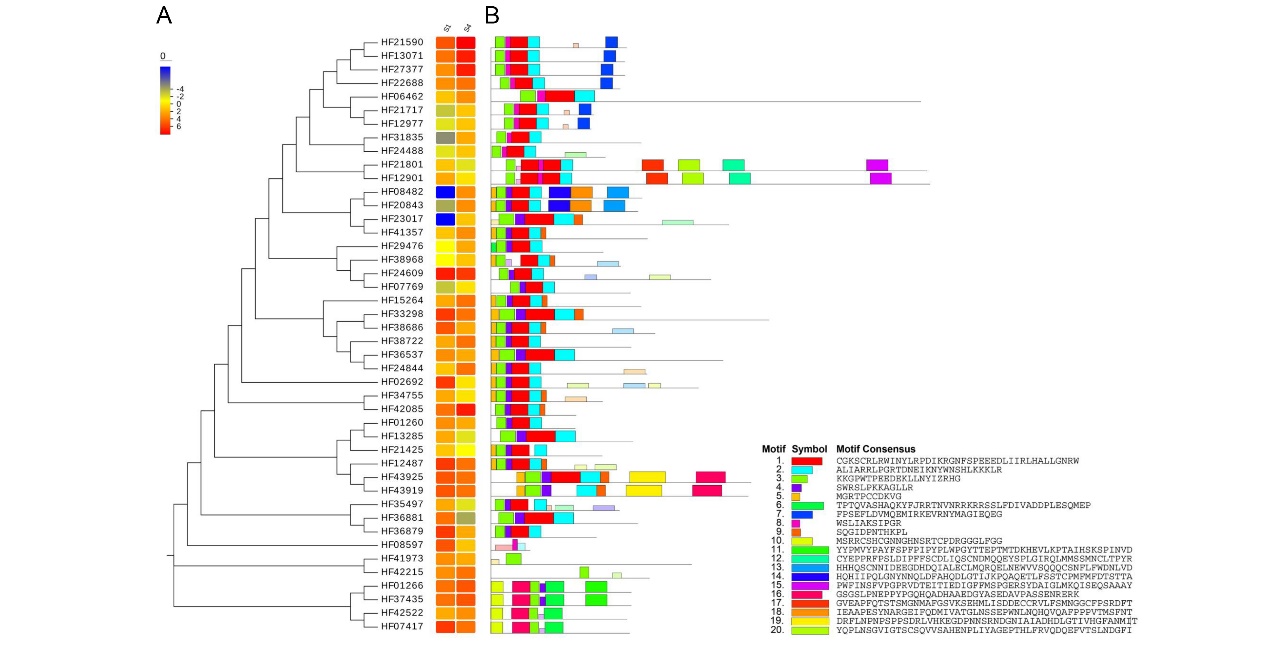


**Supplementary Figure 1**. Motif composition and MYB DNA-binding domains of differentially expressed MYB transcription factors (TFs) in S1 and S4. (A) Heat map comparing differentially expressed genes in S1 and S4. (B) Motif composition of 44 MrMYB TFs. Motifs, numbered 1 to 20, are displayed in different colored boxes.


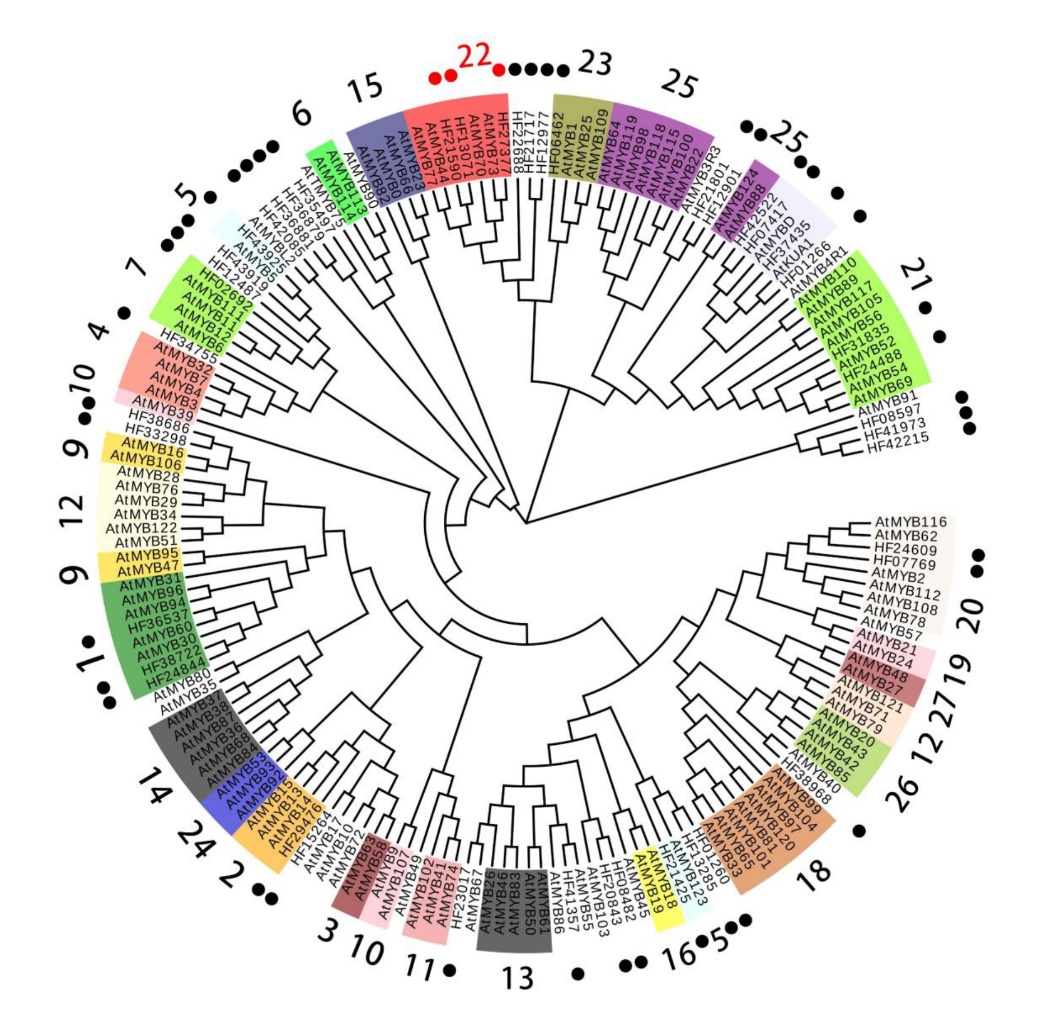


**Supplementary Figure 2.** Evolutionary relations among *MrMYB*s. Full-length amino acid sequences of MYBs from the transcriptome data set and *Arabidopsis* genome were first aligned using ClustalW in MEGA6. The phylogenetic tree was constructed according to the neighbor-joining method. Branches corresponding to partitions reproduced in less than 50% of the bootstrap replicates were collapsed. Three *MrMYB44-like*s were marked with red dots and other 41 *MrMYB44-like*s were identified from RNA-seq data of *M*alus ‘Radiant’. Arabic numerals outside the circle indicate gene families.


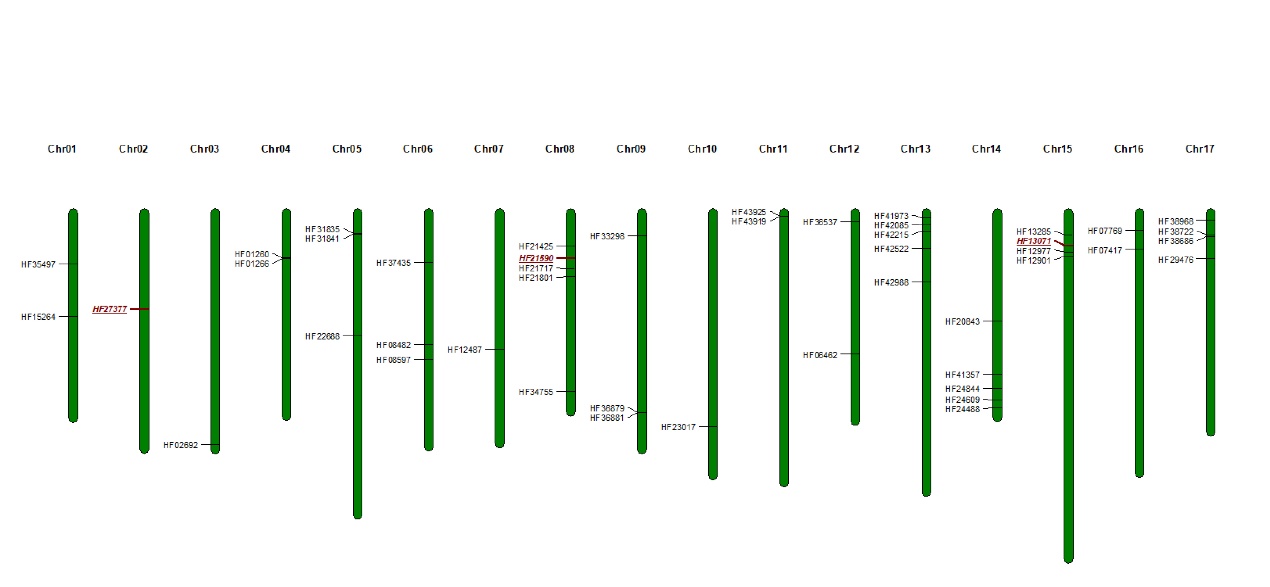


**Supplementary Figure 3.** Chromosomal location of differentially expressed MYB transcription factors in *Malus* ‘Radiant’. *Malus* × *domestica* HFTH1 Whole Genome version 1.0 was used to conduct chromosomal location. The red genes are *MrMYB44-like1* (HF13071) located in chromosome 15, *MrMYB44-like2* (HF21590) located in chromosome 8, *MrMYB44-like3* (HF27377) located in chromosome 2, respectively.


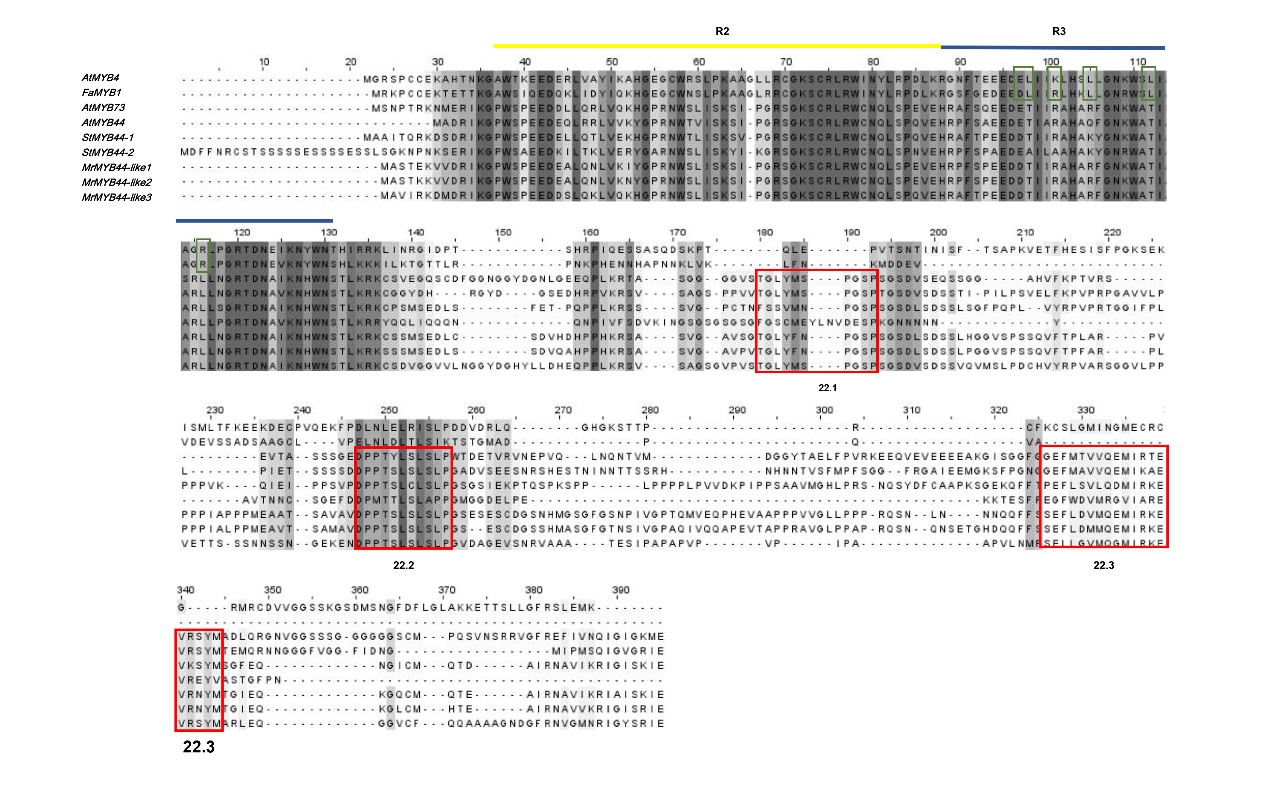


**Supplementary Figure 4.** Amino acid sequence alignment of *MrMYB44-like1*, *MrMYB44-like2*, *MrMYB44-like3*, and other known anthocyanin-related repressors. Conserved residues and partially conserved residues are shown in black. R2 and R3 domains are indicated with yellow and blue lines, respectively. Conserved motifs 22.1 (TGLYMSPxSP), 22.2 (D/EPP/MTxLxLSLP), and 22.3 (GxFMxVVQEMIxxEVRSYM) in the C-terminus of SG22 are shown in red boxes. Conserved bHLH-interacting motifs are shown in green boxes. The following genes were used with their GenBank or Arabidopsis TAIR accession numbers: *Arabidopsis thaliana* *AtMYB4* (AT4G38620), *AtMYB73* (AT4G37260), *AtMYB44* (AT5G67300), *Fagaria ananassa* *FaMYB1* (AF401220.1), and *Solanum tuberosum* *StMYB44-1* (MK410941.1) and *StMYB44-2* (MK410942.1).


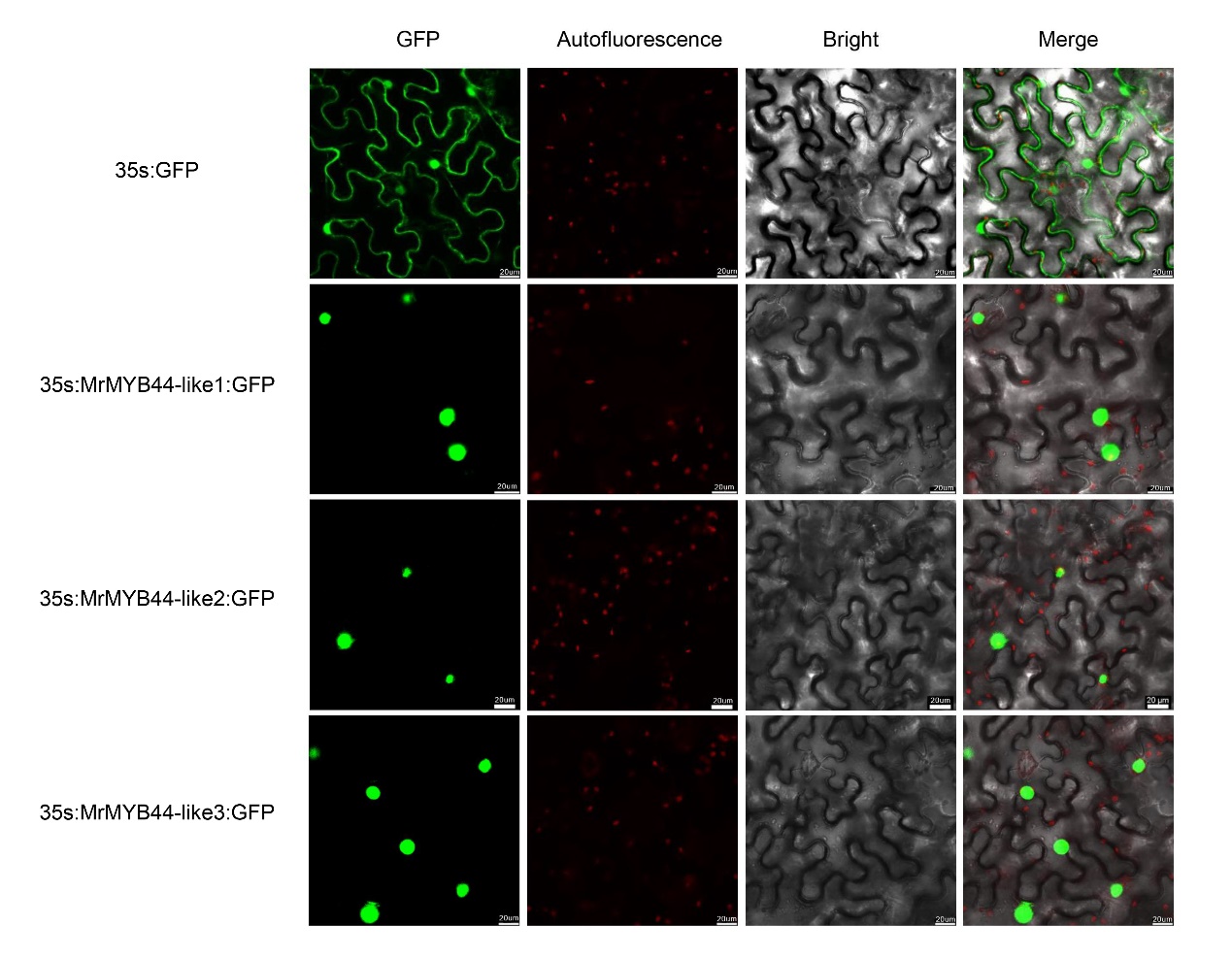


**Supplementary Figure 5.** Subcellular localization of *MrMYB44-like1/2/3*. Transient transformation of *Nicotiana* *benthamiana* leaves with 35S: GFP or 35S:*MrMYB44-like1/2/3*: GFP fusion proteins revealed that *MrMYB44-like1/2/3* are located in the nucleus. Autofluorescence: chloroplast autofluorescence; GFP: GFP fluorescence; Merge: merged images of chloroplast autofluorescence, GFP fluorescence, and bright-field microscopy. Bars, 20 μm.
